# Stable isotope data (oxygen-18 and deuterium) from surveys of lakes, wetlands, rivers, and input waters across the South Athabasca Oil Sands region, Alberta, 2007–2009

**DOI:** 10.1016/j.dib.2018.12.074

**Published:** 2018-12-31

**Authors:** J.J. Gibson, S.J. Birks, M.C. Moncur

**Affiliations:** a InnoTech Alberta, 3-4476 Markham Street, Victoria, BC, Canada V8Z 7X8; b University of Victoria, Department of Geography, Victoria, BC, Canada V8W 3R4; c InnoTech Alberta, 3608-33 St NW, Calgary, AB, Canada T2L 2A6

## Abstract

Oxygen-18 and deuterium analyses of water samples are provided from a regional survey of lakes, wetlands, soil waters, groundwaters, and snowpack samples collected in the Southern Athabasca Oil Sands (SAOS) region, Alberta, Canada, mainly during 2007–2009. Lake, wetland, and river sampling were conducted by helicopter during late summer, capturing conditions close to peak evaporative enrichment. Shallow soil water from the unsaturated zone was also collected in late summer, whereas deeper groundwaters from Quaternary aquifers, Quaternary channels, and uppermost Cretaceous strata, were collected primarily as part of winter drilling programs by industrial partners. Snowpack samples were collected in late March/early April, prior to significant spring melt. This dataset includes 1576 isotopic analyses made on 788 water samples as well as selected isotope mass balance model outputs (lake evaporation/inflow and water yield to lakes). These basic model data are provided to facilitate evaluation of the method as a tool for spatial mapping of water yield and its interannual variability. Details and further discussion on the isotope mass balance approach are provided in “Mapping water yield distribution across the southern Athabasca Oil Sands area: baseline surveys applying isotope mass balance of lakes” (Gibson et al., 2019). Overall, the data are expected to be useful, in comparison with local and regional datasets, for water resource management and planning, including design of monitoring networks and environmental impact assessments for oil sands projects.

**Specifications table**

**Table:** 

Subject area	*Water resources, hydrology, hydrogeology*
More specific subject area	*Stable isotope tracers*
Type of data	*Table, figure, .xlsx file*
How data were acquired	*Isotope ratio mass spectrometry,Thermofisher Scientific, Delta V with Gasbench (oxygen-18) and H-Device (for deuterium). Excel was used for isotope mass balance modelling. ArcGIS and ArcHYDRO were used for spatial analysis.*
Data format	*Isotope analytical data are reported in per mil relative to Vienna Standard Mean Ocean Water (‰ VSMOW) and normalized to SMOW/SLAP (Standard Light Antarctic Precipitation); evaporation/inflow ratios are reported as percentages (evaporation/inflow X 100%); water yield to lakes is reported in millimetres per year (mm/year).*
Experimental factors	*Water samples were collected in tightly-sealed 30* *mL HDPE bottles and stored at room temperature prior to analysis; Spatially representative climate data (temperature, relative humidity, precipitation and evaporation) were obtained from a regional re-analysis product; lake and watershed areas were delineated from a 30-m digital elevation model; isotope balance calculations were based on a commonly-used model*[1].
Experimental features	*Water samples were collected by float plane, or by helicopter, the latter by hovering over the centre of the lake and lowering a bucket to collect a near-surface sample (at approx. 0.5-m depth). Wetland samples were collected at the approximate centre of the nearest neighboring wetland to selected study lakes. River samples were collected by helicopter and targeted the mouths of major rivers, as well as upstream reaches, mainly above geological transitions. Soil samples were collected along highways by digging a pit to maximum depth of 1-m in a representative forested area adjacent to the highway. Water was extracted from soil using azeotropic distillation with toluene. Snow was collected in snow pits, whereby a representative sample of the entire snowpack was collected, placed in a plastic bag and allowed to fully melt at room temperature prior to transferring to HDPE bottles. Groundwater was collected from industrial wells using various standard pumping methods.*
Data source location	*Southern Athabasca Oil Sands Region, a 35,000* *km*^*2*^*wetland rich boreal forest region south of Fort McMurray, Alberta, Canada.*
Data accessibility	*Data are located in this article*
Related research article	Gibson, J.J., Birks, S.J., Moncur, M.C., Mapping water yield distribution across the southern Athabasca Oil Sands area: baseline surveys applying isotope mass balance of lakes. Journal of Hydrology: Regional Studies 21 (2019), 1–13, https://doi.org/10.1016/j.ejrh.2018.11.001[1].

**Value of the data**

• This is a benchmark survey, as sampling was conducted prior to significant in situ oil sands development in the region, although seismic and gas development precedes our survey. It is also unique as a large number of lakes, wetlands, and rivers were resampled in several consecutive years.
• The isotopic data and model outputs offer new insight into water cycling processes, spatial and temporal variability in surface water balance, and potential for surface/groundwater interactions.
• Isotope mass balance model outputs for lakes may be explored as an approach to map water yield at a higher spatial resolution than conventional hydrometric monitoring networks. Repeat future surveys may be useful for characterizing impacts due to industrial development.
• Isotopic and model data may be useful for designing monitoring programs, to ensure that the full range of water budget conditions are characterized.

## 1 Data

**Fig. 1:**
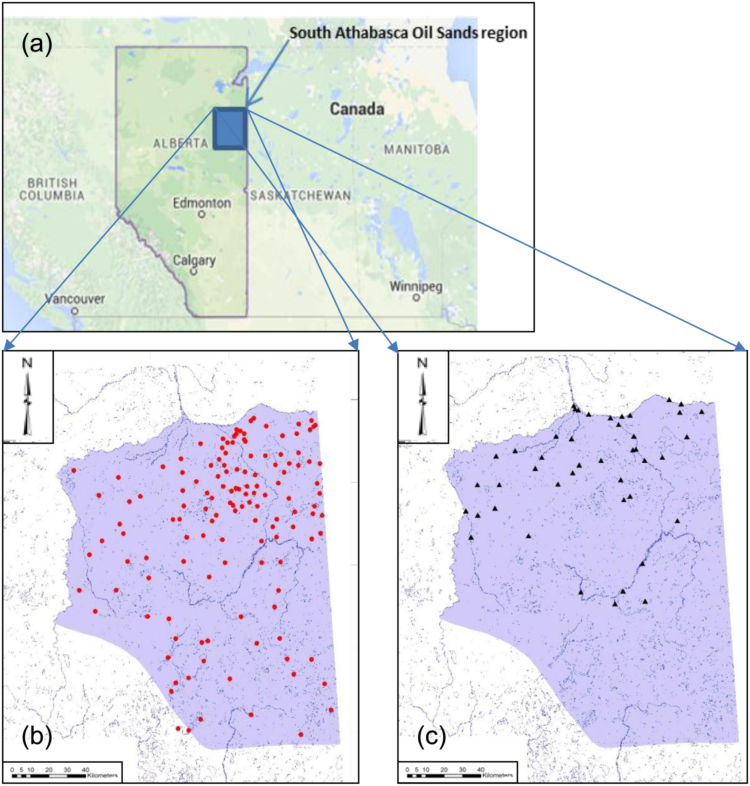
Location of (a) Southern Athabasca Oil Sands (SAOS) region within Alberta, Canada; (b) lakes and (c) rivers sampled in the SAOS in 2007, and repeat sampled in 2008 and 2009.

Stable isotope data (oxygen-18 and deuterium) for water samples are provided, including lakes, wetlands, rivers, soil porewater, groundwater, and snowpack analyzed following collection as part of field programs during 2007 to 2009 across the south Athabasca region (see Fig. 1, Table 1, SAOSswdata.xlsx). Water sampling and analysis was supported by InnoTech Alberta and its predecessors, and several industrial partners. Sampling was conducted in late summer/early fall, as has been recommended for regional stable isotope surveys of lakes in cold regions [2]. For reference, snowpack data are included from 2012 snow surveys in late March/April. Model outputs, including lake evaporation/inflow ratios and water yield are provided based on a simple isotope mass balance model assuming well-mixed water bodies [1]. The method involves estimation of evaporation/inflow for each lake based on characterization of the isotopic composition of lake water and precipitation input, and utilizes basic climate data for each site (evaporation, precipitation, humidity, temperature) as well as calculated values for the isotopic composition of atmospheric moisture based on a partial equilibrium model [3]. Calculated inputs to the lake that are not accounted for by precipitation on the lake surface are presumed to be due to water yield from the catchment area. Note that nearly identical approaches have been used to estimate water balance parameters and water yield in a wide variety of published assessments across Canada, including application to assess critical loads of acidity to lakes within Canada׳s Acid Rain program [4], [5], [6], [7], [8], [9].

**Table 1:** Lake size distribution and number of lakes selected.

***Size class***	***1***	***2***	***3***	***4***	***5***	***6***	***7***	***8***	***Total***
***Area*****(km^2^)**	*>50*	*5*–*50*	*1.0*–*5.0*	*0.5–1.0*	*0.1*–*0.5*	*0.05*–*0.1*	*0.02*–*0.05*	*0.01*–*0.02*	
***No. lakes sampled***	*3*	*22*	*33*	*18*	*33*	*5*	*5*	*9*	*128*
Total No. lakes in reference area*(51,295** km**^**2**^***)***	*3*	*24*	*91*	*119*	*683*	*446*	*817*	*1631*	*3814*
***Percentage sampled***	*100*	*92*	*36*	*15*	*5*	*1.1*	*0.6*	*0.6*	*3.4*

* Includes 27,945 km^2^ in SAOS Alberta and 23,350 km^2^ in adjacent regions of Saskatchewan.

## 2 Experimental design, materials and methods

### 2.1 Site selection

Prior to field work, lakes were selected for water sampling based on a stratified, random sampling framework designed to provide a representative selection of lakes across the region. The study domain initally included ~28,000 km^2^ within the SAOS area, as well as an additional area of ~24,000 km^2^ in adjacent Saskatechewan, the latter of which was not sampled as part of this survey. Lake areas and perimeters were used to determine lake classes for lakes located within the study domain using a pre-existing approach [10]. Similar methods have been used by researchers in Canada׳s Acid Rain Program for critical loadings assessment [11]. The general approach for selecting lakes was to target lakes from a representative range of classes (Table 1). Further selection considered the need for distributed areal coverage, surficial geology, subcrop, drainage patterns, and proximity to the oil sands properties.

In previous regional lake surveys designed to examine acidification, all lakes in class 1 and 50% of the lakes in class 2 were selected with remaining resources divided in proportion to average class distribution ratios, weighted according to lake density variations. All of the larger lakes were similarly selected (i.e. falling in class 1 and 2) within the Alberta portion of the study area, while an attempt was made to survey special interest zones for industry within and around the Christina River development corridor. Table 1 shows the classes and number of lakes selected for each class. A broad distribution of lakes was sampled, especially within classes 2–5 (i.e. lakes ranging in size from 0.1–50 km^2^). Lakes in the very smallest classes (i.e. classes 6–8) are numerous in the study area and less than 1% of these lakes were included. Overall, approximately 12% of the lakes within the SAOS in classes 1 through 5 were sampled. A map showing the resulting distribution of lake sampling sites across the region suggests slightly higher density of sampling points in the northeastern quadrant of the SAOS where lake density is higher, although coverage extends across the entire region (Fig. 1).

### 2.2 Water sampling and analysis

Water samples for analysis of the stable isotopes of water were collected in order to establish water balance conditions in lakes using an isotope balance method [1], and to better understand spatial and temporal variability in surface water budgets across the region, both at present and in preparation for comparison with a series of repeat surveys to be carried out periodically over the next several decades to assess response to progressive stages of in situ oil sands development. Such information has also been applied along with water quality data, radon-222, tritium, and an extended array of solute isotopes [12] to evaluate biogeochemical cycling, critical loads of acidity, and surface groundwater interaction.

Water samples were analyzed for oxygen-18 and deuterium using established methods ([13], [14], respectively). Samples are reported in “δ” notation in per mil (‰) relative to Vienna Standard Mean Ocean Water (V-SMOW), and were normalized on the SMOW/SLAP scale [15].

### 2.3 Model data outputs

Lake and watershed areas were delineated using ArcGIS and ArcHYDRO based on a 30-m resolution digital elevation model (www.geobase.ca). Climatological parameters near ground surface (i.e. precipitation, temperature, relative humidity, evaporation and precipitation) were obtained by interpolation from the North American Regional Reanalysis dataset, consistent with an approach described previously [7]. Isotopic composition of input to lakes was estimated from the intercept of the local meteoric water line and the local evaporation line [1], and isotopic composition of atmospheric moisture was assessed using the partial equilibrium approach [3], which involved fitting predicted oxygen-18 and deuterium enrichment to match the slope of the local evaporation line [1]; see also [16]. A detailed description of the model has been provided in [1].
